# Detection and Characterisation of The ‘A’ Variant of Rabbit Haemorrhagic Disease (RHDVa, GI.1a) Over a Decade (2013–2023) in Italy

**DOI:** 10.1155/tbed/8762378

**Published:** 2026-09-30

**Authors:** Vittoria Di Giovanni, Moira Bazzucchi, Lorenzo Capucci, Antonio Lavazza, Patrizia Cavadini

**Affiliations:** ^1^ Istituto Zooprofilattico Sperimentale della Lombardia e dell’Emilia-Romagna ‘Bruno Ubertini’ (IZSLER) WOAH Reference Laboratory for Rabbit Haemorrhagic Disease Brescia 25124 Italy izsler.it

## Abstract

Since the 1980s, highly pathogenic and hepatotropic *Lagovirus* species have significantly impacted rabbit populations, including Rabbit Haemorrhagic Disease Virus (RHDV, genotype GI.1), its variant RHDVa (genotype GI.1a), and RHDV type 2 (RHDV2, genotype GI.2). Non‐pathogenic *Lagovirus* strains such as Rabbit Calicivirus (RCV, genotypes GI.3 and GI.4), which mainly replicate in the gastrointestinal tract, have also been identified. Recombination between pathogenic and non‐pathogenic strains, particularly at the junction between non‐structural (NS) and structural genome regions, is a major driver of the emergence of new variant strains. In the context of the widespread and predominant circulation of RHDV2, we analysed 51 RHDVa strains collected from domestic rabbit farms across Italy between 2013 and 2023 to assess their genetic and antigenic variability. Phylogenetic analyses were performed using full and partial genome sequences, and antigenic characterisation was conducted using a panel of RHDVa‐specific monoclonal antibodies (MAbs). Phylogenetic analysis showed no evidence of recombination involving the Italian RHDVa strains. Nucleotide and amino acid identities with the reference strain RHDVa_Pv97 ranged from 95% to 98% and 97%–99%, respectively, confirming high genetic stability. The antigenic study indicated overall stability, consistent with the genetic results, but the few mutants found allowed us to map at least two epitopes located on the outer loops of the VP60 protein, which constituted a large part of the virus surface. In particular, the substitution at position N414S leads to the loss of MAb 2B4 binding to the RHDV/Italy/FG2014_1 strain, and the N348S mutation, which is present in five strains, results in the acquisition of binding to the MAb 2A10. Despite the global predominance of RHDV2, RHDVa persists to be detected sporadically in various Italian regions, especially within commercial rabbit farms. Continuous molecular and antigenic monitoring of *Lagovirus* strains remains essential to detect potential co‐circulation and recombination events that could lead to the emergence of novel strains with distinct genomic and antigenic traits.

## 1. Introduction

Rabbit haemorrhagic disease (RHD), one of the major threats to the health of both domestic and wild adult rabbits (*Oryctolagus cuniculus*), is a highly contagious viral disease first identified in China in 1984 [1, 2]. RHD causes fatal hepatitis and diffuse haemorrhages, notably affecting the lungs, heart, kidneys, and spleen enlargement, leading to death within 24–48 h post‐infection [3]. Since its emergence, RHD has spread rapidly worldwide, quickly becoming endemic in many regions. Indeed, it has an ecological impact, causing massive declines in many wild rabbit populations and adversely affecting their dependent predators in Mediterranean ecosystems [4], as well as an economic impact, causing considerable losses to the rabbit farming industry, especially in countries such as Italy, Spain, France and China, where most of the rabbit meat industry is concentrated. Additionally, in 1996, RHDV was introduced into Australia as a biological control agent in the long‐standing effort to manage wild rabbit populations, an invasive alien species responsible for serious ecological degradation and significant damage to agriculture [5].

The aetiological agent, RHD virus (RHDV), is a positive‐sense, single‐stranded RNA virus classified within the genus *Lagovirus* of the family *Caliciviridae* [6, 7].

The RHDV genome is ~7.5 kb and organised into two overlapping open reading frames (ORFs). ORF 1 contains seven non‐structural (NS) genes upstream of the capsid protein (VP60), while the 3^′^‐terminal ORF2 encodes a minor structural protein (VP10) [8].

According to the phylogenetic classification proposed by Le Pendu et al. [9], the genus *Lagovirus* comprises several genotypes. RHDV corresponds to genotype GI.1, while a novel related virus that emerged in France in 2010, named RHDV2, has been assigned to genotype GI.2 [10, 11]. The lagovirus that affects the hare and causes the deadly disease European Brown Hare Syndrome (EBHSV) [12] is classified as genotype GII. In addition, the genus *Lagovirus* also includes non‐pathogenic viruses. Rabbit Caliciviruses (RCV/GI.3‐GI.4) were first identified in domestic and wild rabbits in Italy in 1996 [13] and then in France [14, 15], in Australia in 2000 [16], and very recently also in Chile [17]. They differ significantly from RHDV as they have intestinal tropism and share a low genomic identity (about 92%) with the VP60 capsid gene of RHDV. Similarly, in hares, a non‐pathogenic lagovirus (HaCV) classified as GII.2 has recently been described in Europe [18–21] and Australia [22].

In 1997, the first “field” antigenic variant of RHDV, named RHDVa (genotype GI.1a), was identified almost simultaneously on rabbit farms in Italy and Germany [23, 24]. In subsequent years, the RHDVa variant became widespread in Italy and was also reported in other European countries, as well as in Asia, the Americas, and Australia [25–33]. However, retrospective evidence indicates that viruses belonging to this lineage were already present in China in 1985 (GenBank: AY269825.1), consistent with the estimated mean date of the “Most Recent Common Ancestor” of RHDVa around 1966 [34]. These data suggest that RHDVa likely first emerged in China and subsequently spread to Europe, where it progressively replaced classical RHDV strains, possibly reflecting a selective advantage, as shown in Italy, where in 2003, 81% of Italian RHD outbreaks were associated with RHDVa (Capucci and Lavazza, personal communication).

RHDVa was characterised by the lack of reactivity with monoclonal antibodies (MAbs) developed against the classical RHDV strain Bs89, particularly the 1H8 Mab [23, 35]. Genetic analyses revealed about 93% nucleotide identity between RHDV and RHDVa VP60 genes, with the main antigenic changes associated with amino acid substitutions in the hypervariable region spanning residues 344–370 of the VP60 protein [23, 36].

RHDV2 marks a significant step forward in the evolution of pathogenic Lagoviruses, dramatically changing their epidemiology since it emerged in 2010 in France. Although it is genetically related to RHDV—with an average nucleotide identity of 82.4% based on VP60 sequences—this virus forms a new serotype capable of infecting rabbits that have been vaccinated against RHDV. It can also cause disease in animals only a few weeks old [10, 11] and shows a broader host range that includes other lagomorph species [35, 37–41]. Thanks to these biological traits and the naïve status of wild rabbit populations towards this new virus, RHDV2 has spread rapidly worldwide, nearly replacing classical RHDV strains. However, the original RHDV and especially its variant, RHDVa, have still been sporadically detected in intensive and backyard farms as well as in wild rabbit populations [33, 42].

The simultaneous circulation of multiple *Lagovirus* genotypes, often within the same geographical area and time period, has facilitated the occurrence of recombination events—one of the key evolutionary mechanisms in *Lagovirus* [43–45]. In this genus and other members of the *Caliciviridae* family, recombination typically occurs at the junction between NS and structural genes, resulting in the emergence of new viral strains with unique genomic compositions [46–49]. The novel virus, RHDV2, is believed to have originated from a recombination event between a non‐pathogenic virus (GI.3), which provided the NS portion of the genome, and an unknown virus that contributed the region encoding the VP60 capsid protein [48]. Moreover, recombinant strains of RHDV have been identified in Australia, in which the structural portion of the genome derives from RHDVa (GI.1a), whereas the NS genes are most closely related to those of non‐pathogenic RCV [33].

In this study, we analysed 51 RHDVa strains collected and identified at IZSLER from ~685 RHD cases diagnosed in Italy between 2013 and 2023. They were primarily sourced from domestic rabbit farms. We evaluated the genetic variability of this genotype over time, investigated the occurrence of recombinant strains, and assessed antigenic variations using a panel of MAbs previously raised specifically against RHDVa and used in antigenic studies [28, 35].

Our findings indicate that RHDVa is quite stable at the genetic and antigenic levels, and, by analysing seven mutated strains with mAbs, we revealed only minor variations and localised two distinct epitopes on VP60.

## 2. Materials and Methods

### 2.1. Samples and Epidemiological Data

As the Italian reference centre for viral diseases affecting lagomorphs, we collected liver samples from rabbits that, as part of the routine diagnostic workflow, tested positive for RHDVa by virological ELISA [50]. The samples were collected starting from 2013 onwards across various Italian regions. They originated from different types of rabbits, including rabbits reared in intensive farms for commercial production, from small rural/familiar units, from domestic “pet” rabbits, as well as from free‐living wild/feral rabbits (Table 1). Additionally, one sample was obtained from a rabbit farm located in Malta because of its close geographic proximity and the existing common exchanges and movements of animals, feeding and equipment, which could favour cross‐border virus transmission between the two countries. In Table 1, we report the month/year of collection, strain identification, the code number used to identify each strain (GenBank accession number), the region, province and municipality of origin and the geographic location, as shown in Figure 1.

**Table 1:** Summary of the data of the RHDVa samples analysed in this study.

Date	Strain identification (GenBank#)	Region	Prov.	Municipality	Type of farm	Map #
Feb‐13	RHDV/Italy/BS2013_1 PX448258	Lombardy	BS	Botticino	Backyard/familiar farm	1
Nov‐13	RHDV/Italy/BS2013_2 PX448303	Lombardy	BS	Lograto	Backyard/familiar farm	2
Jan‐14	RHDV/Italy/BS2014_1 PX448259	Lombardy	BS	Nuvolera	Backyard/familiar farm	3
Feb‐14	RHDV/Italy/BS2014_2 PX448260	Lombardy	BS	Ospitaletto	Intensive commercial	4
Apr‐14	RHDV/Italy/BS2014_3 PX448261	Lombardy	BS	Bagnolo Mella	Backyard/familiar farm	5
May‐14	RHDV/Italy/BS2014_4 PX448301	Lombardy	BS	Villa Carcina	Backyard/familiar farm	6
Jun‐14	RHDV/Italy/BS2014_5 PX448262	Lombardy	BS	Caino	Backyard/familiar farm	7
Aug‐14	RHDV/Italy/FG2014_1 PX448302	Puglia	FG	Volturara Appula	Intensive commercial	20
Aug‐14	RHDV/Italy/FG2014_2 PX448273	Puglia	FG	Volturara Appula	Intensive commercial	21
Dec‐14	RHDV/Malta/2014 PX448274	//	EE	Marsa	Intensive commercial	22
Jan‐15	RHDV/Italy/BS2015_1 PX448263	Lombardy	BS	Bagnolo Mella	Backyard/familiar farm	8
Feb‐15	RHDV/Italy/LT2015 PX448307	Lazio	LT	Latina	Backyard/familiar farm	28
Mar‐15	RHDV/Italy/MI2015 PX448275	Lombardy	MI	Milano	Pet rabbit	23
Apr‐15	RHDV/Italy/BS2015_3 PX448306	Lombardy	BS	Caino	Backyard/familiar farm	10
May‐15	RHDV/Italy/TV2015 PX448278	Veneto	TV	Treviso	Backyard/familiar farm	26
Jun‐15	RHDV/Italy/BS2015_2 PX448264	Lombardy	BS	Ossimo	Backyard/familiar farm	9
Jun‐15	RHDV/Italy/BG2015 PX448300	Lombardy	BG	Albino	Backyard/familiar farm	24
Nov‐15	RHDV/Italy/NA2015 PX448279	Campania	NA	Giugliano	Backyard/familiar farm	27
Jan‐16	RHDV/Italy/BS2016_1 PX448265	Lombardy	BS	Bovezzo	Backyard/familiar farm	11
Feb‐16	RHDV/Italy/RA2016_1 PX448280	Emilia‐Romagna	RA	Lugo	Wild/feral rabbit	33
Apr‐16	RHDV/Italy/RA2016_2 PX448281	Emilia‐Romagna	RA	Fusignano	Backyard/familiar farm	34
Aug‐16	RHDV/Italy/BS2016_2 PX448266	Lombardy	BS	Concesio	Backyard/familiar farm	12
Oct‐16	RHDV/Italy/BS2016_3 PX448267	Lombardy	BS	Manerbio	Backyard/familiar farm	13
Oct‐16	RHDV/Italy/PO2016_1 PX448283	Tuscany	PO	Prato	Backyard/familiar farm	37
Oct‐16	RHDV/Italy/RI2016_1 PX448304	Lazio	RI	Rieti	Backyard/familiar farm	29
Nov‐16	RHDV/Italy/RI2016_2 PX448305	Lazio	RI	Greccio	Backyard/familiar farm	30
Dec‐16	RHDV/Italy/Lazio2016 PX448299	Lazio	RI	Rieti	Backyard/familiar farm	32
Dec‐16	RHDV/Italy/RE2016 PX448288	Emilia‐Romagna	RE	Reggio nell’Emilia	Wild/feral rabbit	40
Dec‐16	RHDV/Italy/PO2016_2 PX448284	Tuscany	PO	Prato	Backyard/familiar farm	38
Dec‐16	RHDV/Italy/FI2016 PX448277	Tuscany	FI	ND	Backyard/familiar farm	39
Jan‐17	RHDV/Italy/BS2017_1 PX448268	Lombardy	BS	Nuvolera	Backyard/familiar farm	14
Feb‐17	RHDV/Italy/BS2017_2 PX448269	Lombardy	BS	Mazzano	Backyard/familiar farm	15
Feb‐17	RHDV/Italy/MO2017_1 PX448295	Emilia‐Romagna	MO	Pavullo nel Frignano	Backyard/familiar farm	43
Mar‐17	RHDV/Italy/RA2017_1 PX448282	Emilia‐Romagna	RA	Ravenna	Wild/feral rabbit	35
Apr‐17	RHDV/Italy/Molise2017 PX448297	Molise	IS	Cerro al Volturno	Backyard/familiar farm	45
May‐17	RHDV/Italy/BS2017_3 PX448270	Lombardy	BS	Verolanuova	Backyard/familiar farm	16
May‐17	RHDV/Italy/MO2017_2 PX448296	Emilia‐Romagna	MO	Pavullo nel Frignano	Backyard/familiar farm	44
May‐17	RHDV/Italy/RA2017_2 PX448298	Emilia‐Romagna	RA	Faenza	Intensive commercial	36
Oct‐17	RHDV/Italy/RI2017 PX448276	Lazio	RI	Rieti	Backyard/familiar farm	31
Mar‐18	RHDV/Italy/RM2018 PX448285	Lazio	RI	Grotti Cittaducale	Backyard/familiar farm	46
Oct‐18	RHDV/Italy/TO2018 PX448286	Piemonte	TO	ND	Pet rabbit	47
Nov‐18	RHDV/Italy/BS2018 PX448287	Lombardy	BS	Corte Franca	Backyard/familiar farm	17
Jan‐19	RHDV/Italy/RE2019_1 PX448289	Emilia‐Romagna	RE	Reggio nell’Emilia	Backyard/familiar farm	41
Mar‐19	RHDV/Italy/RE2019_2 PX448290	Emilia‐Romagna	RE	Reggio nell’Emilia	Wild/feral rabbit	42
May‐19	RHDV/Italy/Marche2019 PX448291	Marche	AN	ND	Backyard/familiar farm	50
May‐19	RHDV/Italy/BS2019 PX448271	Lombardy	BS	Brescia	Backyard/familiar farm	18
May‐19	RHDV/Italy/BG2019 PX448292	Lombardy	BG	Almè	Backyard/familiar farm	25
Sep‐19	RHDV/Italy/PR2019 PX448293	Emilia‐Romagna	PR	Fidenza	Backyard/familiar farm	48
Jan‐20	RHDV/Italy/BL2020 PX448294	Veneto	BL	Tambre	Backyard/familiar farm	49
Sep‐20	RHDV/Italy/BS2020 PX448272	Lombardy	BS	Darfo Boario Terme	Backyard/familiar farm	19
May‐23	RHDV/Italy/AN2023 PX448308	Marche	AN	Monte San Vito	Backyard/familiar farm	51

**Figure 1:**
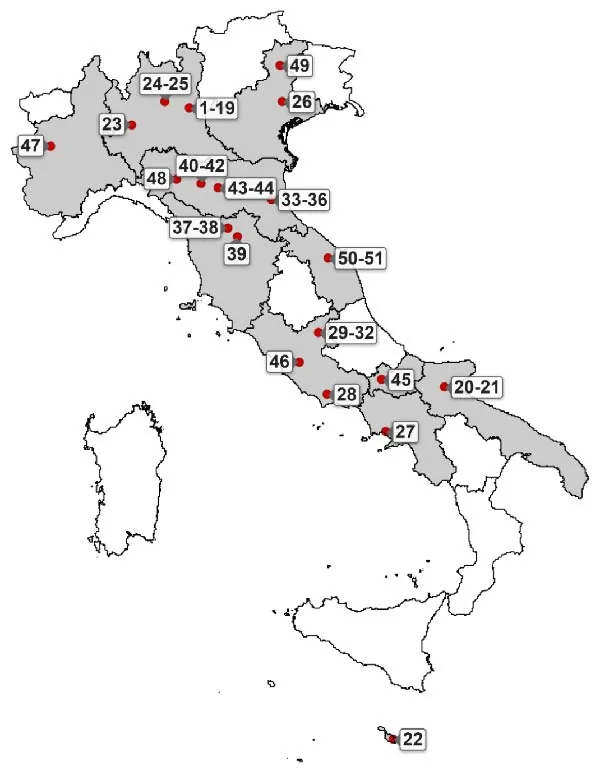
The map displays the sequenced strains, along with their identification number and origin. Each strain is marked at its geographic location.

Italy is one of the leading countries producing rabbit meat in the world and has over 1000 intensive farms with almost 3,000,000 does. Whereas any movements of rabbits introduced in these farms (mainly restocking females) and exiting the farm to the slaughterhouse and of people and vehicles are all registered according to national and European rules, vice versa, it is always very difficult to get anamnestic data concerning small family units (less than 50 breeding rabbits), which are indeed very numerous and spread in almost all the territory, since these are not under the control of official authorities. Therefore, in these small family units, we got some information just after the outbreak occurred. In addition, since they were run by people without technical training who knew very little about the disease, they had not used the RHD vaccine as a preventive measure. On the contrary, vaccination for RHD is a common practice in commercial farms: breeding rabbits are vaccinated with an inactivated, adjuvanted vaccine every 6 months, while fattening growing rabbits are not vaccinated, considering the quite short production life (they are slaughtered at 70–75 days of age, and till 40–45 days of age are protected by maternal passive antibodies). Since around 2000, commercially available vaccines have included bivalent RHDV + RHDVa and, since 2013, monovalent RHDV2 vaccines. The bivalent vaccine RHDV + RHDVa was commonly used until around 2015–2016, but as it became clear that RHDV2 was becoming the predominant strain causing RHD, it was gradually phased out in favour of vaccines specifically designed for RHDV2.

Regardless of the type of farming, we always observed similar numbers for morbidity (about 100%) and mortality (up to 80%–90%). In fact, in those cases that occurred in familiar units, it was precisely the very high mortality that induced the owner to look for a laboratory diagnosis. In intensive farms, when outbreaks of the disease occurred, the “high” mortality was limited to growing non‐vaccinated rabbits since breeding does were mostly protected by antibodies induced by vaccination.

### 2.2. RNA Extraction and cDNA Synthesis

Liver samples were homogenised at 10% (w/v) in phosphate‐buffered saline (PBS; 10 mM phosphate buffer, 150 mM NaCl, pH 7.4) using an Ultraturrax homogeniser (IKA‐Werke GmbH & Co., Staufen, Germany). The homogenates were centrifuged at 5000 × *g* for 10 min at 4°C, and the resulting supernatants were stored at −20°C with 50% glycerol added. Twenty microlitres were used for RNA extraction using the TRIzol reagent (Qiagen, Hilden, Germany), according to the manufacturer’s instructions.

First‐strand cDNA synthesis was performed using an oligo(dT) adapter (5^′^‐GACTCGAGTCGACATCGATTTTTTTTTTTTTTTTT‐3^′^) [51] and the SuperScript IV Reverse Transcriptase kit (Thermo Fisher Scientific, Waltham, MA, USA).

### 2.3. Genome Sequencing

Viral genome amplification was performed by PCR using TaKaRa LA Taq DNA Polymerase (Takara Bio Inc., Shiga, Japan) and primers designed for the *Lagovirus* genome (Table 2). The amplified products were gel‐purified (Macherey‐Nagel, Düren, Germany), and libraries were prepared using the Illumina DNA Prep (M) Tagmentation Kit (Illumina Inc., San Diego, CA, USA) according to the manufacturer’s standard protocol. Sequencing was carried out on the Illumina MiniSeq platform in the paired‐end mode (2 × 150 bp). Raw reads were analysed using Geneious Prime v.2022.2.1 (Biomatters Ltd., Auckland, New Zealand), and resulting sequences were mapped against the reference genome EF558581 (RHDVa) obtained from the Genbank.

**Table 2:** Primers used for the amplification of the genome of RHDVa strains: the nucleotide positions are based on the RHDV strain GenBank accession X87607.

Primer	Sequence 5^′^‐3^′^	Position nt	References
Primers for amplification of Vp60 gene including a portion of RdP			
4420 F	GTYGGCRTTGACATGACMTC	4420–4439	[49]
109 Rev	ACTACTAGCGTGGTCACCACC	7044–7066	[11]
Primers for full genome amplification (ORF1+ORF2)			
RHDV‐24‐F	GTGAAAGTTATGGCGGCTATGTCG	1–24	[52]
Adapter	GACTCGAGTCGACATCG	—	[51]

### 2.4. Phylogenetic and Recombination Analysis

Two datasets of *Lagovirus* sequences (including the strains from this study) were used for phylogenetic analysis: one comprising 117 full‐genome sequences and another comprising 146 VP60 sequences because more RHDVa sequences are available only for the genome’s structural region (Table S1). The selected sequences, available in GenBank, represent a subset of the main *Lagovirus* genotypes (identified based on the VP60 sequence). In particular, sequences identified through BLAST analysis against the strains characterised in this study (NS and VP60 genes, Table S2) were selected and increased to include representative, non‐redundant sequences for each recognised genotype to ensure a balanced spatial and temporal distribution of the included strains: GI.1 (29), GI.1a (52), GI.3 (4), GI.4 (4), GI.5 (1) [52], GI.2 (4) and GII.1 as the outgroup (GenBank KC832838). Sequence alignment was performed with MEGA X [53], and analyses were conducted separately for the NS gene region (1–5222 nt) and structural gene VP60 (5223–6851 nt). The best‐fitting nucleotide substitution model was determined in MEGA X using the Bayesian Information Criterion (BIC). The GTR + G+I model showed the lowest BIC score and was therefore selected for subsequent phylogenetic analyses. The proportion of trees in which related taxa clustered together is shown next to each branch. Initial trees for the heuristic search were automatically generated by applying the neighbour‐joining method to a matrix of pairwise distances estimated using the Maximum Composite Likelihood (MCL) approach, and the topology with the highest log‐likelihood score was chosen. The tree was scaled, with branch lengths representing the number of substitutions per site. Clade support was assessed using 1000 bootstrap replicates.

To further assess the robustness of the phylogenetic inference performed with MEGA X, additional maximum‐likelihood analyses were performed using IQ‐TREE [54] through the Galaxy Europe platform (Phylogenomic/evolutionary tree construction from multiple sequences, Galaxy Version 2.4.0+galaxy2). Phylogenetic relationships among the sequences included in the full genome dataset were inferred using the Maximum Likelihood (ML) method implemented in IQ‐TREE v2.4.0. For each multiple sequence alignment, the best‐fitting nucleotide substitution model was selected using ModelFinder according to the Akaike Information Criterion (AIC) [55]. The selected models were GTR + F+I+R4 for the 6,851‐nt alignment, SYM + I+R4 for the 5222‐nt alignment, and GTR + F+I+R3 for the 1629‐nt alignment. ML phylogenies were reconstructed using the corresponding substitution models. Branch support was assessed using 1000 ultrafast jackknife replicates implemented in IQ‐TREE. Trees were inferred as unrooted, with sequence KC832839_EBHSV displayed as the outgroup for graphical representation only. IQ‐TREE analyses were performed using default settings, with automatic model selection by ModelFinder and branch support estimated by ultrafast jackknife. The resulting topologies were compared with those obtained using MEGA X.

In addition, Bayesian phylogenetic analyses were carried out using the BEAST v2.7.7 [56]. Separate analyses were performed for the 6851‐nt alignment, for the 5222‐nt alignment, and for the 1629‐nt alignment under a Bayesian Skyline coalescent model and a strict molecular clock. Markov chain Monte Carlo (MCMC) chains were run for 10 million generations, with parameters and trees sampled every 1000 generations. Nucleotide substitutions were modelled using a GTR model with gamma‐distributed rate heterogeneity among sites (five categories). Posterior tree distributions were subsequently summarised to infer phylogenetic relationships among lagovirus strains.

Furthermore, a dataset of full‐genome sequences was screened for potential recombination events involving RHDVa sequences using RDP4 (version 4.100) [57] and SimPlot [58]. Six methods implemented in RDP4 were used (RDP window size 60, Bootscan window size 500, MaxChi window size 120, Chimaera window size 120, SiScan window size 500 and GENECOV with default settings), with the following parameters: sequences were set to linear, Bonferroni correction, highest acceptable *p*‐value of 0.05, and 500 permutations. Only recombination events supported by at least three independent methods were considered significant. Similarity plot and BootScan analyses were independently performed in SimPlot using the Kimura two‐parameter model, a sliding window of 200 bp, a step size of 20 bp, and 1000 bootstrap replicates.

In addition, the HyPhy Genetic Algorithm for Recombination Detection (GARD; analysis version 0.2) [59] implemented through the Galaxy platform (HyPhy version 2.5.96+galaxy0) was applied. GARD uses a genetic algorithm‐based heuristic approach to identify phylogenetic incongruence among different alignment partitions and compares alternative models with increasing numbers of recombination breakpoints using the corrected AIC (c‐AIC).

The analysis was performed on a nucleotide alignment of full genome sequences using gamma‐distributed rate variation with five rate classes. The maximum number of breakpoints considered was set to 10,000, and the analysis was performed using the default normal search mode. The algorithm first evaluated a single‐partition model without recombination breakpoints, followed by stepwise breakpoint searches. The best‐supported model was selected according to the lowest c‐AIC value. Breakpoint‐associated topological incongruence was subsequently evaluated by comparing the phylogenetic trees inferred for the different genomic partitions. Recombination was considered biologically relevant only when breakpoint‐associated topological changes involved the Italian RHDVa (GI.1a) sequences analysed in this study.

### 2.5. Antigenic Analysis of RHDVa Strains

The antigenic profile of 51 strains was determined using a panel of specific MAbs produced against both RHDVa (strain Pv97) and RHDV (strain Bs89) (GenBank Accession numbers EU250330 and ×87607, respectively), which were previously used also for RHDV2 antigenic characterisation [35]. The MAbs were employed in a sandwich ELISA as previously described by Lopes et al. [60], with rabbit anti‐RHDVa immune serum adsorbed onto the plate, followed by the addition of the MAbs at a concentration of ~100 ng/mL. Conjugated anti‐mouse rabbit IgG was used as the secondary tracer antibody. Each sample was tested in duplicate three times. For each MAb, the absorbance values at 492 nm were converted to a percentage of reactivity, using as 100% the mean value of all samples with an OD falling within a range of ±0.12 OD (3 standard deviations) relative to that of the reference strain RHDVa_Pv97. Given that the coefficient of variation in the percentage reactivity of RHDVa strains across days was ≤10, we classified the reactivity of the strains with each MAb into three categories: positive (≥70%), intermediate (>15% and <70%), and negative (<15%). These threshold values were determined empirically, based on the authors’ laboratory experience, taking into account the inherent biological variability of the results obtained when crude liver extracts were used as antigens in the ELISA assay. Indeed, the reactivity of each individual sample may vary depending on its quality (e.g., partial capsid degradation and differing ratios of capsids to 6S subunits) [61], which in turn depends primarily on the degree of viral replication in the liver and the time elapsed since the rabbit’s death, as well as on the collection and storage conditions applied before testing.

In addition, to better characterise the binding properties of the 5 RHDVa strains with an estimated positive reactivity with 2A10 (reappearance on RHDVa of a specific RHDV epitope), additional ELISA tests were performed. These assays followed the sandwich ELISA setup described above, except for the MAbs’ concentrations: the initial concentration was set at 1 µg/mL, followed by two‐fold serial dilutions. Strains RHDVa_Pv97, RHDV_Bs89, and RHDV/Italy/BS2013_2 served as controls for the ELISA assays performed. The final results of the ELISA tests are presented graphically in the relevant section.

## 3. Results

### 3.1. Phylogenetic Analysis

A total of 51 nucleotide sequences were obtained from either partial or near‐complete viral genome sequencing (Table 1). Forty‐five sequences spanned nearly the entire genome, although they lacked the 5^′^‐untranslated region, resulting in ~15 nucleotides shorter than the full‐genome sequences. The total length of the sequenced gRNA was 7354 bp. The remaining six partial sequences consisted solely of the VP60 gene. The overall genome matched that of other *Lagovirus* strains, featuring two overlapping ORFs: ORF1 (nucleotides 1–7020) and ORF2 (nucleotides 7001–7354).

VP60 sequences were compared with the Italian reference strain RHDVa_Pv97 (GenBank accession no. EU250330) using BLAST analysis, revealing nucleotide identities between 95% and 98% and amino acid identities between 97% and 99%. Phylogenetic analysis was performed separately for sequences encoding NS proteins and VP60 because recombination frequently occurs at the junction between the NS and structural genes in Lagoviruses [43]; therefore, phylogenetic analyses based only on whole‐genome sequences may lead to misleading inferences [62].

To further assess the robustness of the phylogenetic inference performed with MEGA X (Figure 2), additional ML (Figure S3) and Bayesian phylogenetic analyses (Figure S4) performed independently on three genomic datasets (full genome, NS and VP60) produced highly congruent topologies, with consistent placement of all Italian strains across trees.

Figure 2Maximum Likelihood (ML) phylogenetic trees inferred using MEGA X for: (A) the structural gene VP60 (data set of 146 VP60 sequences) (nucleotides 5223–6851; substitution model GTR + G + I), and (B) the non‐structural genes (data set of 117 full‐genome sequences) (nucleotides 1–5222; substitution model GTR + G + I). Trees are drawn to scale, with branch lengths representing the number of substitutions per site. Node support was assessed using 1000 bootstrap replicates, and bootstrap values above 70% are shown. Genotype clusters that did not include virus variants sequenced in this study were collapsed and annotated accordingly. European brown hare syndrome virus (EBHSV) was used to root the tree (GenBank Accession No. KC832838).

**Figure:**
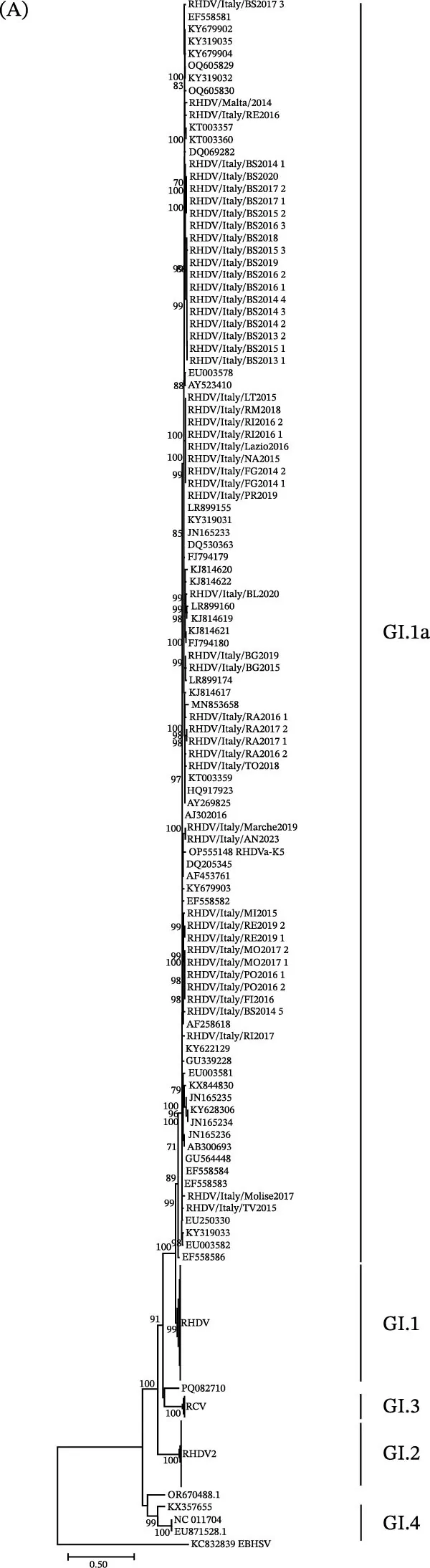

**Figure:**
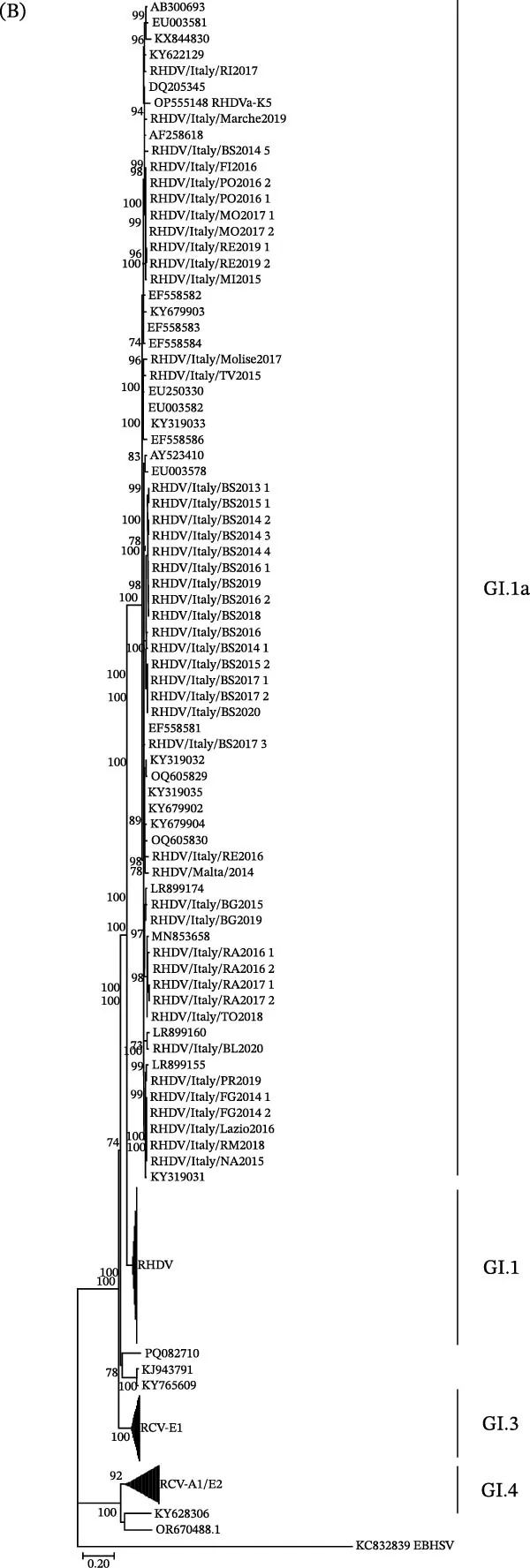

We additionally performed a BLAST analysis of the 51 sequences described in this study, using all sequences present in GenBank (accessed on 1 July 2026, https://blast.ncbi.nlm.nih.gov/Blast.cgi) [63], considering separately the genomic regions encoding the NS and structural proteins. The results of these analyses are reported in Table S2.

#### 3.1.1. VP60 Phylogeny

The phylogenetic trees based on VP60 sequences showed that all strains detected in various Italian regions, as well as the one from Malta, clustered within the GI.1a (RHDVa) lineage together with representative reference strains, which also include strains from larger geographical regions (Europe, Australia, Asia and America), confirming their classification within the RHDVa variant. Italian sequences did not form a single monophyletic group but were distributed among several well‐supported clusters. In particular, strains collected in the Brescia province between 2013 and 2020 consistently grouped within the same highly supported cluster, suggesting the long‐term persistence of a closely related viral lineage (Figure 2A and Figures S3 and S4). Nucleotide BLAST analysis showed the lowest identity between sequences RHDV/Italy/BS2015_3 and RHDV/Italy/BS2017_1 (97.3%), while the highest was between RHDV/Italy/BS2014_3 and RHDV/Italy/BS2014_2 (99.88%), suggesting they were the same virus. Other samples from Brescia Province were more heterogeneously distributed within the broader RHDVa clade.

Within the phylogenetic tree, additional clusters were identified, including samples not necessarily originating from the same province, as clearly observed in the case of Brescia Province, but also from more distant geographical areas. For example, a cluster includes five samples from the provinces of Turin (TO) and Ravenna (RA): the first is from north‐western Italy, while the other four are from north‐east Italy. In particular, the four Ravenna province sequences (RHDV/Italy/RA2016_1, RHDV/Italy/RA2016_2, RHDV/Italy/RA2017_1, RHDV/Italy/RA2017_2) showed an average nucleotide identity of 98.72%.

Two other clusters included samples collected from neighbouring regions or, in some cases, from the same province but at slightly different times. One cluster contains eight samples from central and southern Italy that are highly similar. Within this cluster, the highest identity (99.94%) was observed between two sequences (RHDV/Italy/FG2014_1 and RHDV/Italy/FG2014_2) collected from the same farm 2 months apart, indicating the virus persisted on the farm. Another cluster includes samples from Tuscany and Emilia‐Romagna, with two pairs of sequences showing 100% nucleotide identity. One pair corresponds to samples from the provinces of Florence and Prato (Tuscany) (RHDV/Italy/FI2016 and RHDV/Italy/PO2016_2), collected in the same month and year. The second pair comes from the province of Modena (Emilia‐Romagna), gathered 3 months apart from the same municipality (RHDV/Italy/MO2017_1 and RHDV/Italy/MO2017_2). Since the latter samples originated from the same municipality but from different farms at a 3‐month interval, a common source could be inferred based on this level of similarity. This cluster also included two additional samples from Lombardy (Brescia and Milano provinces: RHDV/Italy/BS2014_5 and RHDV/Italy/MI2015), two others from Emilia‐Romagna (RHDV/Italy/RE2019_1 and RHDV/Italy/RE2019_2), and another one from Tuscany (RHDV/Italy/PO2016_1).

As previously mentioned, the strain from Malta (RHDV/Malta/2014) clustered within the RHDVa clade, showing nucleotide identity values ranging from 93.48% with sample RHDV/Italy/Molise2017 to 97.54% with sample RHDV/Italy/RE2016, which also clustered within the same phylogenetic branch.

#### 3.1.2. NS Phylogeny and No Evidence of Recombination Within the Italian Strains

The results of the phylogenetic analysis of the NS part of the genome (Figure 2B and Figures S3 and S4) are comparable with those obtained for VP60, all showing that the Italian strains cluster within the RHDVa clade, except for samples where the NS region could not be sequenced and were therefore excluded from the analysis.

This phylogenetic concordance, together with the absence of statistically supported recombination signals detected using RDP4, SimPlot and GARD (data not shown), indicates that none of the Italian RHDVa strains analysed in this study shows evidence of recombinant origin. In detail, GARD analysis identified evidence of recombination‐compatible signals within the complete dataset. The single‐partition model without breakpoints showed a c‐AIC value of 181,944.67, whereas the inclusion of recombination breakpoints substantially improved the model fit. The best‐supported model included two breakpoints located at nucleotide positions 1749 and 5246 (c‐AIC = 176,471.30). A model including three breakpoints (positions 900, 3532, and 5235) resulted in a higher c‐AIC value (176,596.49) and was therefore not selected as the preferred model. The GARD‐defined partitions corresponded to three genomic regions (nt positions 1–1749, 1750–5246, and 5247–6851). Although the topological incongruence test supported the presence of at least one partition with a different evolutionary history at the dataset level, comparison of the phylogenetic trees reconstructed for the three genomic regions did not reveal conflicting placements involving the Italian RHDVa sequences. The Italian strains consistently retained the same phylogenetic relationships across all partitions. Therefore, the detected breakpoint signals are unlikely to represent recombination events affecting the Italian RHDVa lineage circulating between 2013 and 2023.

### 3.2. Antigenic Study

The antigenic study of 48 strains revealed four antigenic profiles (A, B, C and D) based on differences in reactivity with 12 MAbs; however, a profile could not be assigned to three samples because the virions were present in a ’smooth’ form, lacking the outer half of the VP60 protein [64]. This structural alteration, confirmed by Western blotting (data not shown), resulted in an atypical antigenic profile.

Figure 3 presents the representative strains for each profile. Profile A, which includes the RHDVa reference strain Pv97, was the most common (85.4%). This profile is characterised by reactivity with the RHDVa‐specific MAbs, as well as with the RHDV cross‐reactive MAbs 6F9 and 6H6, which recognise epitopes shared between RHDV and RHDVa [35] and shows no reactivity with the other RHDV‐specific MAbs. The strains in this group were collected over different years and in different locations.

**Figure 3:**
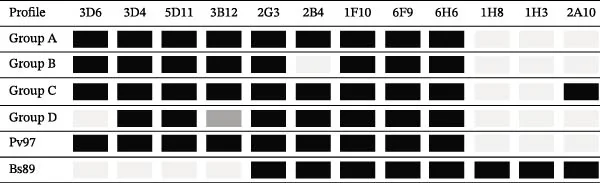
Antigenic profiles identified using the panel of 12 monoclonal antibodies. RHDVa strains are divided into groups A through D based on their reactivity with MAbs. Black rectangles—full reactivity; dark grey rectangle—moderate reactivity; light grey rectangles—no reactivity. A: 41 samples, B: 1 sample (RHDV/Italy/FG2014_1), C: 5 samples (RHDV/Italy: BS2015_2, BS2016_3, BS2017_1, BS2017_2, BS2020) and D: 1 sample (RHDV/Italy/NA2015). The RHDVa (Pv97) and RHDV (Bs89) reference strains are shown. MAbs from 3D6 to 1F10 were raised against RHDVa_Pv97; from 6F9 to 2A10 were raised against RHDV_Bs89.

#### 3.2.1. RHDVa Strains That Have Lost Some of Their Original Epitopes

Profiles B and D were represented by a single strain each: RHDV/Italy/FG2014_1 and RHDV/Italy/NA2015, respectively. These samples differ from profile A because they do not bind to a single RHDVa‐specific MAb. More precisely, sample RHDV/Italy/FG2014_1 did not react with MAb 2B4, while sample RHDV/Italy/NA2015 showed no reactivity with MAb 3D6. The loss of reactivity in these two MAbs could be due to epitope modification, probably caused by the replacement of one or more amino acids critical for the epitope’s conformation [60, 65]. The sequence comparison of the analysed samples suggests that the loss of MAb 2B4 binding is likely linked to the N414S mutation, which is found only in the RHDV/Italy/FG2014_1 sample (Figure 4A). Similarly, the lack of reactivity of MAb 3D6 may be related to the A369S mutation present only in the RHDV/Italy/NA2015 sample. In both cases, these amino acids are located in the P2 domain, specifically in two of the seven variable regions described by Wang et al. [36], namely, regions V3 and V5, well exposed on the capsid surface (Figure 4B).

**Figure 4:**
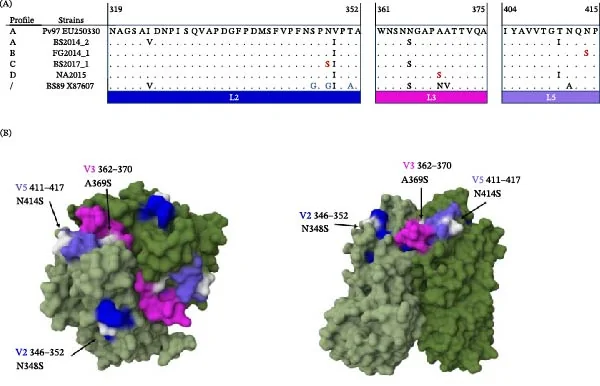
Structural mapping of MAbs’ epitopes. (A) RHDVa amino acid alignment of VP60 loops (L2, L3 and L5) of the strains belonging to the four profiles. The mutations identified in the three strains (Profiles B–D) are shown in red compared to the Italian reference strain RHDVa_Pv97. (B) Structural representation of RHDVa P dimers (Protein Data Bank code 4EGT– molecular surface), chain A (light green) and chain B (dark green), shown from top and side views. Variable (V) regions within different loops [36] are depicted in the same colours (blue, magenta and violet) and as the sequences shown (A). Only those affected by mutations (V2, V3, V5) are reported. Critical amino acids within the variable regions are coloured white and marked with the corresponding mutation. Mol ^∗^ 3D Viewer (https://www.rcsb.org/3d-view) was used to modify the dimers.

#### 3.2.2. RHDVa Strains that Have Acquired a Specific RHDV Epitope

The situation differs for profile C, identified in five samples (10.4%), all from the Brescia province (RHDV/Italy: BS2015_2, BS2016_3, BS2017_1, BS2017_2 and BS2020), although collected in different months and years. Conversely, this profile is characterised by the ‘re‐emergence’ of a specific RHDV epitope recognised by MAb 2A10. This is most likely linked to the N348S mutation—the only substitution shared by all five samples—located within the V2 variable region of the P2 domain (Figure 4A). It should be noted that the amino acid sequence encompassing position 348 is 343‐PFNGPGIPAA‐352 in RHDV_Bs89, whereas in RHDVa strains reactive with MAb 2A10, it is 343‐PFNSPSIPTA‐352. The two sequences differ by three amino acids (highlighted in blue in Figure 4A), demonstrating that the MAb is able to bind non‐identical epitopes. Position 348 is critical to MAb 2A10 binding: Glycine (G) in RHDV_Bs89 and Serine (S) in the five mutated RHDVa allow binding, whereas Asparagine (N), present in all other RHDVa strains, abolishes it, likely due to its bulkier and more complex side chain (Figure 4B).

#### 3.2.3. Binding Properties of MAb 2A10 in Profile C Samples

To explore whether the three–amino acid divergence (shown in blue in Figure 4A) between the five strains of profile C and the RHDV_Bs89 reference strain might influence MAb 2A10 binding properties, a comparative ELISA was performed. The assay included the five RHDVa samples, together with RHDV_Bs89, RHDVa_Pv97 and BS2013_2 as reference strains. In addition to MAb 2A10, a representative subset of MAbs from the original panel was used: MAb 5D11 as an RHDVa‐specific positive control; MAb 2G3 for its known cross‐reactivity between RHDV and RHDVa; and MAb 1H3, an RHDV‐specific antibody, as a negative control for RHDVa reactivity.

As shown in Figure 5, MAbs 5D11 and 2G3 displayed comparable reactivity across all RHDVa samples, with nearly overlapping titration curves, while MAb 1H3 remained consistently negative. Regarding MAb 2A10, the response curves of the 2A10‐positive samples appeared to differ visually from that of strain RHDV_Bs89, suggesting that amino acid differences in loop 2 may contribute to the observed patterns. Notably, sample BS2016_3 exhibited a response profile consistent with that observed for RHDV_Bs89. In this assay, both samples reached an OD_492_ value of 1.0 at nearly the same concentrations (~15 and 30 ng/mL, respectively), differing by only a single twofold dilution step. In contrast, although the other four RHDVa samples showed consistent reactivity with their specific antibodies, their 2A10 titration curves exhibited a steeper decline in the signal. Consequently, these samples reached the OD_492_ threshold of 1.0 at significantly higher concentrations (~62–125 ng/mL) compared to the 15–30 ng/mL range observed for BS2016_3. This deviation, relative to the specific MAbs, may indicate a reduced binding efficiency in these four RHDVa samples.

**Figure 5:**
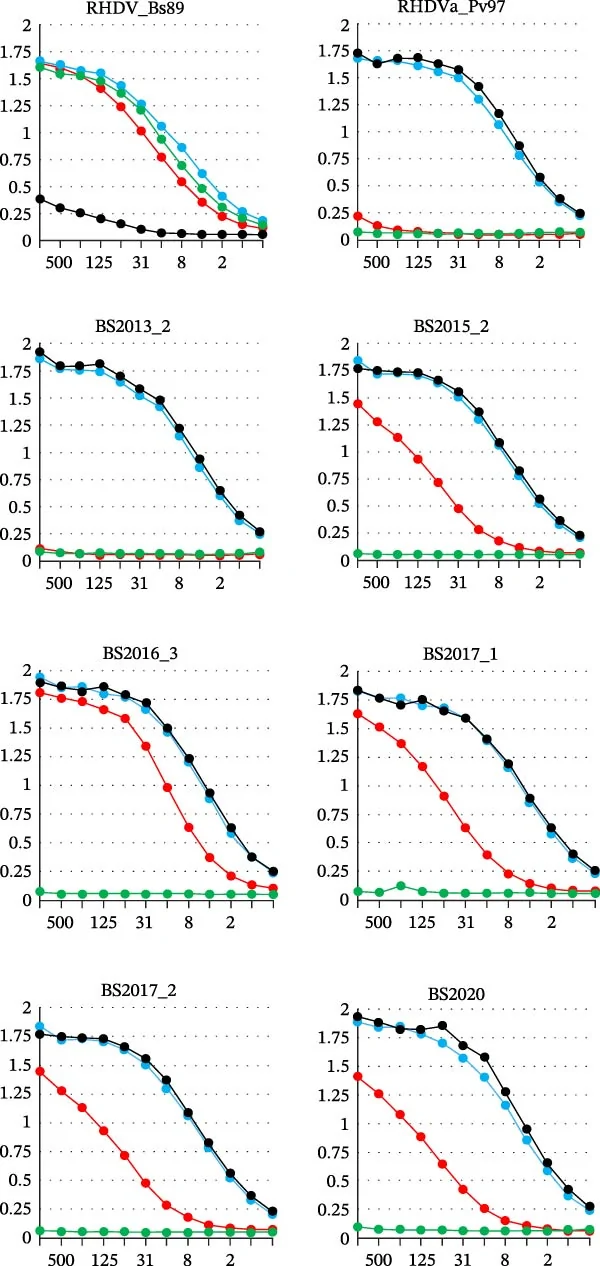
Reactivity profiles of MAbs 2A10 (red), 2G3 (blue), 5D11 (black), and 1H3 (green) against RHDV reference strain Bs89, RHDVa reference strain Pv97, Profile A sample BS2013_2, and Profile C samples. The *x*‐axis represents the MAb concentration in ng/ml (two‐fold serial dilutions), while the *y*‐axis shows the optical density values at 492 nm.

To further investigate potential structural factors underlying the variability in MAb 2A10 reactivity among these five samples, amino acid sequence analysis was extended to loops structurally adjacent to loop 2, where the N348S substitution was identified. As shown in Figure 6A, the four samples displaying low 2A10 reactivity (P++) carry the N430S mutation within loop 6. Furthermore, while residue 430 is located 82 positions downstream of residue 348 in the VP60 primary sequence, protein folding brings these two positions into proximity on the viral surface (Figure 6B). This structural arrangement, combined with the fact that the high‐reactivity sample BS2016_3 (P+++) retains the ancestral asparagine found in the RHDV_Bs89 reference strain, suggests that position 430 is a key component of the 2A10 epitope and that an asparagine at this site is critical for optimal binding efficiency.

**Figure 6:**
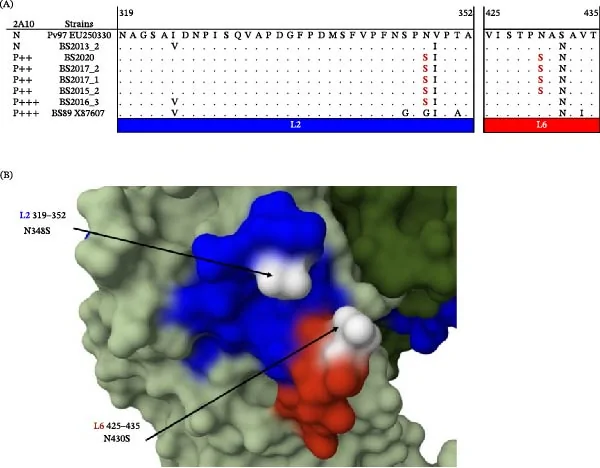
(A) RHDVa amino acid alignment of VP60 (loops 2 and 6). All strains of profile C, one of profile A, and the references are reported. Amino acids potentially involved in the different binding affinity with MAb 2A10 are highlighted in red. 2A10 reactivity is indicated as follows: *N* = negative, P++ = low, P+++ = high. (B) Detailed representation of the spatial proximity between these two loops within the P2 domain RHDVa (Protein Data Bank code 4EGT– molecular surface). Residues at positions 348 (loop 2 in blue) and 430 (loop 6 in purple) are highlighted in white.

## 4. Discussion

Since its emergence in France in 2010, RHDV2 (RHDV2/GI.2) has gradually replaced the original RHDV (GI.1) worldwide, especially in areas with wild lagomorph populations (e.g., the Iberian Peninsula, France, North America and Australia). Although research has mainly focused on the evolutionary history of RHDV2 (GI.2) from its emergence to the present, it remains worthwhile to examine the presence of RHDVa (GI.1a) in livestock, which appears not to have been entirely displaced by RHDV2. In Italy, despite RHDV2 (GI.2) causing most of the RHD outbreaks reported annually, as in other parts of the world, the antigenic variant RHDVa (GI.1a), first identified in 1997, continues to be found sporadically in the country, mainly on industrial and backyard rabbit farms.

The reason why RHDVa has not fully disappeared and continues to re‐emerge sporadically remains unclear, and we have no precise explanations other than speculative hypotheses. The sporadic reappearance of RHDVa, mainly in small rural farms, at least according to our data, despite its almost complete displacement by RHDV2 in lagomorphs, suggests that there could be some factors that might help explain its persistence, also beyond direct viral competition, such as differences in host susceptibility or distinct environmental pressures associated with livestock farming [32]. As a matter of fact, rural rabbit farms, such as the one linked to the most recent RHDVa case in the province of Ancona—where 25 out of 30 rabbits died, with no other outbreaks reported nearby—display distinctive epidemiological dynamics with respect to intensive farmed rabbits, which normally apply stringent biosecurity measures. These farms are characterised by the lack of routine vaccination, minimal to no preventive measures, and frequent opportunities for both direct and, more often, indirect contact with domestic and wild rabbits, often through contaminated hay or other natural feed sources. Indeed, as with RHDV2 outbreaks [66], insects might also serve as passive vectors for the spread of RHDVa. Such rural or family‐run settings often source animals from local markets—typically from other small‐scale operations—or occasionally from industrial farms as weaned kits. Alternatively, internal replacement involves selecting and breeding one or two rabbits across successive generations, thereby further influencing the genetic and immunological profiles of these populations. In addition, pet rabbits may increasingly influence the epidemiology of RHD, as partially shown in this study and in a reported case in Poland, where both RHDV strains (RHDVa and classical RHDV) were detected in companion rabbits [67]. Finally, it cannot be entirely ruled out that other mammal species could serve as potential reservoirs of RHDV, considering the detection of RHDVs in some non‐specific vertebrate hosts sympatric to the European wild rabbits in Europe, including rodents [68, 69] and badgers [70].

This study spans approximately 10 years of *Lagovirus* surveillance across several Italian regions, during which the presence and persistence of RHDVa (GI.1a) were systematically examined at both the genetic and antigenic levels. The phylogenetic analysis of 51 RHDVa (GI.1a) samples collected in Italy between 2013 and 2023, mainly in rural farms, showed that all strains are distributed across European, American and Asian lineages, with no clear phylogenetic clustering by time or geographic location. Although viral circulation has been detected in specific areas such as Brescia Province, the phylogenetic distribution of the Italian strains could even suggest the introduction of novel viral strains from other countries, possibly through commercial trade, as reported by McIntosh et al. [28].

Within the VP60 gene‐based phylogenetic tree, small Italian clusters or subclusters can be recognised, comprising samples that do not necessarily originate from the same province. These clusters show nucleotide identities ranging from 95% to 98% and amino acid identities ranging from 97% to 99% compared to the original RHDVa strain (Pv97). The same range of nucleotide identity is also observed among European, Asian, and American strains. In particular, the likely first RHDVa strain ever detected, that is, the Chinese strain identified in 1985 (GenBank AY269825), exhibited a mean nucleotide identity of 94%–98% with the Italian strains. Although it clustered with strains from different Italian regions as well as strains from Russia, China, and Germany, the node defining this cluster received high bootstrap support (98%). However, its exact internal phylogenetic position cannot be determined with certainty due to low bootstrap values at internal nodes, indicating limited confidence in the finer‐scale relationships. Therefore, the current phylogenetic evidence does not allow the Italian strains to be reliably linked to the earliest Chinese isolate nor does it permit conclusions regarding their evolutionary origin.

The genetic variability between Italian strains, detected over the years and across regions, can be explained by several factors, including vaccination, which has played a significant role in limiting the spread of infection in industrial rabbit farms, thereby limiting replication across different hosts and consequently reducing the potential for the emergence of additional mutations. Notably, since 2010, Italy has widely used the bivalent RHDV‐RHDVa vaccine (Mevax 2 by IZO S.r.l, Brescia, Italy). This specific protection against RHDVa (GI.1a) may have limited its evolutionary potential. Other explanations include the reduced evolutionary pressure exerted on a virus that infects a single host species in a relatively stable ecological environment (e.g., closed farms with few spillover events or host shifts) as well as the virus’s optimal adaptation to its host. Similar findings have been reported in Poland, where RHDVa (GI.1a) strains showed a relatively stable genome [42, 71].

As widely recognised, recombination is a well‐established evolutionary mechanism in *Lagovirus* and *Caliciviridae* in general [48]. However, the Italian strains analysed in this study showed no evidence of recombination, and to date, no naturally occurring recombination events of RHDVa have been reported in European strains to date [42, 71], unlike the extensive recombination documented in RHDV2. The only exception is the RHDVa‐Aus (GI.4e‐GI.1a) strain identified in Australia in 2014, which resulted from recombination, whose origin remains unknown, between a non‐pathogenic RCV‐A1‐like and a pathogenic virus, likely introduced as an exotic incursion from China [33, 45].

The relative genetic stability was further supported by the low antigenic variability detected through MAbs against RHDV and RHDVa strains. Due to the conserved structural organisation of the *Caliciviridae* capsid—comprising an inner S domain and external P1 and P2 domains—the most exposed loops of the capsid protein act as the main antigenic determinants. These loops are highly susceptible to genetic variability, as seen in RHDV2 and other RNA viruses, because of the high replication rates that promote increased genetic and antigenic diversity in association with the selective pressure induced by the host immune system [11, 23, 36].

In this study, we identified four slightly distinct antigenic profiles based on the MAbs reactivity in the sandwich ELISA test. The main profile (A) comprises ~85% of Italian strains, including the prototype Italian RHDVa strain Pv97. Only two strains belonged to profiles B and D, respectively, and each differed in that it had lost its reactivity with a single specific MAb.

The comparative analysis of VP60 amino acid sequences deduced from the nucleotide sequences and the reactivity profiles of individual MAbs suggests the presence of a potential epitope. Specifically, the results demonstrated that substitution at position N414S leads to the loss of MAb 2B4 binding to the RHDV/Italy/FG2014_1 strain. An identical amino acid change, along with the subsequent loss of MAb 2B4 reactivity, was previously reported in one additional RHDVa strain identified in northern Italy in 2006 [72] and in two RHDV (GI.1) strains found in Sardinia in 2010 (GenBank: KC595270 and KC345614; [35]). Further supporting the involvement of this mutation, the reference strain RHDV_Bs89—whose cross‐reactivity with MAb 2B4 is illustrated in Figure 3—also carries an asparagine at this position, consistent with all 50 RHDVa sequences included in the dataset.

Concerning the replacement at position A369S of the RHDV/Italy/NA2015 strain as a possible cause of the loss of binding to MAb 3D6, this remains a hypothesis as this is the only mutant available.

It is interesting to note that the 2A10 MAb, which is negative for the majority of RHDVa strains—being originally developed against the classic RHDV_Bs89 and reactive with all RHDV strains—is nonetheless able to react with five RHDVa samples belonging to the same cluster in the Brescia area and phylogenetically closely related to constitute a separate profile (C). The comparison of the amino acid sequences of these strains shows that they all carry a serine (S) at position 348, in contrast to the asparagine (N) present in strains that do not react with this MAb, strongly suggesting a role for this residue in epitope recognition. This interpretation is further supported by the fact that position 348 is highly exposed on the capsid surface as it is located on loop 2 of the VP60 protein (Figure 4).

Notably, the 2A10 epitope in the RHDVa mutant appears to be partially different from that of RHDV_Bs89, which was used as the immunogen for MAb production. Within the short sequence surrounding position 348, two additional residues (positions 346 and 351) are also mutated. However, this observation is not unexpected given the molecular complexity of the interaction between a MAb and its epitope.

In this regard, Leuthold et al. [73] resolved the X‐ray crystal structure of a protruding RHDV2 domain in complex with a specific MAb. Their study demonstrated that at least nine amino acids contribute to the interaction between the Fab fragment and the viral P subdomain. More importantly, our study suggests that antibody interactions can be influenced by residues in distant loops that are structurally close due to protein folding. In fact, the amino acid position 430 of the VP60 sequence in loop 6 appears to play a key role in binding strength: four 2A10‐positive samples with weaker antibody binding share a serine at this site, while one sample, with a stronger antibody binding, shares the same asparagine as the reference strain RHDV_Bs89. Beyond these sites, we evaluated additional amino acid substitutions throughout the VP60 sequence. However, as these mutations were either located in buried structural regions or showed no consistent correlation with binding patterns, they were not considered to have a significant influence on the 2A10 interaction. Consequently, it is reasonable to assume that the 2A10 epitope comprises at least one more amino acid in addition to that at 348. Finally, it should be noted that the ELISA approach used in this study provides only an approximate estimate of MAb–antigen binding. More quantitative approaches, such as biosensor‐based plasmonic techniques, will be required to further characterise this interaction.

## 5. Conclusions

Surveillance is essential because it enables the identification and characterisation of viral strains, thereby tracking the evolution of RHDV over time and the emergence of new variants or entirely new serotypes, including those resulting from recombination. Simultaneously, genetic analysis of strains offers insights into the mechanisms that drive viral spread and distribution within a specific area, not only natural factors such as species susceptibility, the presence of potential mechanical vectors, and the inherent resistance traits of *Lagovirus* but also human‐related factors connected to livestock production, small‐scale farming, and the increasing popularity of rabbits as pets.

Finally, it should be emphasised that the combined approach of genomic sequence analysis and antigenic reactivity studies using MAbs enables us to expand our understanding of *Lagovirus* immunogenicity. This knowledge is also crucial for the development of next‐generation targeted and safe vaccines that enable easy differentiation between vaccinated and infected animals.

## Funding

This study was supported by the Italian Ministry of Health (Grant IZSLER 014/2013RC) to Lorenzo Capucci, and by IZSLER (Grant COMM_CAR_RHDVa) to Vittoria Di Giovanni.

## Ethics Statement

The authors confirm that they have adhered to the journal’s ethical policies, as noted on the journal’s author guidelines page. Ethical approval was not required as the samples were collected from animals that had already died and sent to the laboratory for diagnostic investigations. The sample collection was performed in accordance with international guidelines.

## Conflicts of Interest

The authors declare no conflicts of interest.

## Supporting Information

Additional supporting information can be found online in the Supporting Information section.

## Supporting information


**Supporting Information** Table S1: Dataset of the GenBank sequences used in this study. Table S2: Nucleotide sequence identity obtained by BLAST analysis for each strain. Figure S3: Maximum likelihood (ML) phylogenetic trees using IQ‐TREE. Figure S4: Bayesian phylogenetic trees using BEAST.

## Data Availability

The data that support the findings of this study are openly available in GenBank at https://www.ncbi.nlm.nih.gov/genbank/ under the reference numbers PX448258–PX448308.

## References

[1] Liu S. , Xue H. , Pu B. , and Qian N. , A New Viral Disease in Rabbits, Animal Husbandry and Veterinary Medicine (Xumu yu Shouyi). (1984) 16, 253–255.

[2] Abrantes J. , van der Loo W. , Le Pendu J. , and Esteves P. J. , Rabbit Haemorrhagic Disease (RHD) and Rabbit Haemorrhagic Disease Virus (RHDV): A Review, Veterinary Research. (2012) 43, no. 1, 10.1186/1297-9716-43-12, 12. PMC3331820 22325049

[3] Marcato P. S. , Benazzi C. , and Vecchi G. , et al.Clinical and Pathological Features of Viral Haemorrhagic Disease of Rabbits and the European Brown Hare Syndrome, Revue Scientifique et Technique de l’OIE. (1991) 10, no. 2, 371–392, 10.20506/rst.10.2.560. 1760582

[4] Monterroso P. , Garrote G. , and Serronha A. , et al.Disease-Mediated Bottom-up Regulation: An Emergent Virus Affects a Keystone Prey, and Alters the Dynamics of Trophic Webs, Scientific Reports. (2016) 6, no. 1, 10.1038/srep36072, 36072. PMC5086860 27796353

[5] Mutze G. , Cooke B. , and Alexander P. , The Initial Impact of Rabbit Hemorrhagic Disease on European Rabbit Populations in South Australia, Journal of Wildlife Diseases. (1998) 34, no. 2, 221–227, 10.7589/0090-3558-34.2.221. 9577768

[6] Ohlinger V. F. , Haas B. , Meyers G. , Weiland F. , and Thiel H. J. , Identification and Characterization of the Virus Causing Rabbit Hemorrhagic Disease, Journal of Virology. (1990) 64, no. 7, 3331–3336, 10.1128/jvi.64.7.3331-3336.1990. PMC249571 2352325

[7] Parra F. and Prieto M. , Purification and Characterization of a Calicivirus as the Causative Agent of a Lethal Hemorrhagic Disease in Rabbits, Journal of Virology. (1990) 64, no. 8, 4013–4015, 10.1128/jvi.64.8.4013-4015.1990. PMC249702 2164609

[8] Meyers G. , Wirblich C. , and Thiel H.-J. , Rabbit Hemorrhagic Disease Virus—Molecular Cloning and Nucleotide Sequencing of a Calicivirus Genome, Virology. (1991) 184, no. 2, 664–676, 10.1016/0042-6822(91)90436-F. 1840711

[9] Le Pendu J. , Abrantes J. , and Bertagnoli S. , et al.Proposal for a Unified Classification System and Nomenclature of Lagoviruses, Journal of General Virology. (2017) 98, no. 7, 1658–1666, 10.1099/jgv.0.000840. 28714849

[10] Le Gall-Reculé G. , Zwingelstein F. , and Boucher S. , et al.Detection of a New Variant of Rabbit Haemorrhagic Disease Virus in France, Veterinary Record. (2011) 168, no. 5, 137–138, 10.1136/vr.d697. 21493491

[11] Le Gall-Reculé G. , Lavazza A. , and Marchandeau S. , et al.Emergence of a New Lagovirus Related to Rabbit Haemorrhagic Disease Virus, Veterinary Research. (2013) 44, no. 1, 10.1186/1297-9716-44-81, 81. PMC3848706 24011218

[12] Frölich K. and Lavazza A. , Alves P. C. , Ferrand N. , and Hackländer K. , European Brown Hare Syndrome, Lagomorph Biology, 2008, Springer, Berlin, Heidelberg, 253–261.

[13] Capucci L. , Fusi P. , Lavazza A. , Pacciarini M. L. , and Rossi C. , Detection and Preliminary Characterization of a New Rabbit Calicivirus Related to Rabbit Hemorrhagic Disease Virus but Nonpathogenic, Journal of Virology. (1996) 70, no. 12, 8614–8623, 10.1128/jvi.70.12.8614-8623.1996. PMC190954 8970986

[14] Le Gall-Reculé G. , Zwingelstein F. , and Fages M.-P. , et al.Characterisation of a Non-Pathogenic and Non-Protective Infectious Rabbit Lagovirus Related to RHDV, Virology. (2011) 410, no. 2, 395–402, 10.1016/j.virol.2010.12.001. 21195443

[15] Lemaitre E. , Zwingelstein F. , Marchandeau S. , and Gall-Reculé G. Le , First Complete Genome Sequence of a European Non-Pathogenic Rabbit Calicivirus (lagovirus GI.3), Archives of Virology. (2018) 163, no. 10, 2921–2924, 10.1007/s00705-018-3901-z. PMC6132933 29978262

[16] Strive T. , Wright J. D. , and Robinson A. J. , Identification and Partial Characterisation of a New Lagovirus in Australian Wild Rabbits, Virology. (2009) 384, no. 1, 97–105, 10.1016/j.virol.2008.11.004. 19049842

[17] Smertina E. , Keller L. M. , and Huang N. , et al.First Detection of Benign Rabbit Caliciviruses in Chile, Viruses. (2024) 16, no. 3, 10.3390/v16030439, 439. PMC10974056 38543804

[18] Cavadini P. , Molinari S. , and Pezzoni G. , et al.Identification of a New Non-Pathogenic Lagovirus in Lepus Europeaus, *Proceedings of the Xth (10th) International Congress of Veterinary Virology*, 2015, 76–77.

[19] Droillard C. , First Complete Genome Sequence of a Hare Calicivirus Strain Isolated From *Lepus europaeus* , Microbiology Resource Announcements. (2018) 7, no. 22, 10.1128/mra.01224-18. PMC6284081 30533849

[20] Droillard C. , Lemaitre E. , and Chatel M. , et al.Genetic Diversity and Evolution of Hare Calicivirus (HaCV), a Recently Identified Lagovirus From *Lepus europaeus* , Infection, Genetics and Evolution. (2020) 82, 10.1016/j.meegid.2020.104310, 104310. 32243924

[21] Cavadini P. , Molinari S. , and Merzoni F. , et al.Widespread Occurrence of the Non-Pathogenic Hare Calicivirus (HaCV Lagovirus GII.2) in Captive-Reared and Free-Living Wild Hares in Europe, Transboundary and Emerging Diseases. (2021) 68, no. 2, 509–518, 10.1111/tbed.13706. PMC8247275 32603021

[22] Mahar J. E. , Hall R. N. , and Shi M. , et al.The Discovery of Three New Hare Lagoviruses Reveals Unexplored Viral Diversity in This Genus, Virus Evolution. (2019) 5, no. 1, 10.1093/ve/vez005, vez005. PMC6456799 30997155

[23] Capucci L. , Fallacara F. , Grazioli S. , Lavazza A. , Pacciarini M. L. , and Brocchi E. , A Further Step in the Evolution of Rabbit Hemorrhagic Disease Virus: The Appearance of the First Consistent Antigenic Variant, Virus Research. (1998) 58, no. 1-2, 115–126, 10.1016/S0168-1702(98)00106-3. 9879768

[24] Schirrmeier H. , Reimann I. , Köllner B. , and Granzow H. , Pathogenic, Antigenic and Molecular Properties of Rabbit Haemorrhagic Disease Virus (RHDV) Isolated From Vaccinated Rabbits: Detection and Characterization of Antigenic Variants, Archives of Virology. (1999) 144, no. 4, 719–735, 10.1007/s007050050538. 10365163

[25] Gall-Reculé G. Le , Zwingelstein F. , Laurent S. , de Boisséson C. , Portejoie Y. , and Rasschaert D. , Phylogenetic Analysis of Rabbit Haemorrhagic Disease Virus in France Between 1993 and 2000, and the Characterisation of RHDV Antigenic Variants, Archives of Virology. (2003) 148, no. 1, 65–81, 10.1007/s00705-002-0908-1. 12536296

[26] Matiz K. , Ursu K. , Kecskeméti S. , Bajmócy E. , and Kiss I. , Phylogenetic Analysis of Rabbit Haemorrhagic Disease Virus (RHDV) Strains Isolated Between 1988 and 2003 in Eastern Hungary, Archives of Virology. (2006) 151, no. 8, 1659–1666, 10.1007/s00705-006-0730-2. 16521047

[27] Farnós O. , Rodríguez D. , and Valdés O. , et al.Molecular and Antigenic Characterization of Rabbit Hemorrhagic Disease Virus Isolated in Cuba Indicates a Distinct Antigenic Subtype, Archives of Virology. (2007) 152, no. 6, 1215–1221, 10.1007/s00705-006-0926-5. 17334949

[28] McIntosh M. T. , Behan S. C. , and Mohamed F. M. , et al.A Pandemic Strain of Calicivirus Threatens Rabbit Industries in the Americas, Virology Journal. (2007) 4, no. 1, 10.1186/1743-422X-4-96, 96. PMC2147015 17910765

[29] Tian L. , Liao J. , Li J.-W. , Zhou W.-R. , Zhang X.-L. , and Wang H.-N. , Isolation and Identification of a Non-Haemagglutinating Strain of Rabbit Hemorrhagic Disease Virus From China and Sequence Analysis for the VP60 Gene, Virus Genes. (2007) 35, no. 3, 745–752, 10.1007/s11262-007-0155-3. 17705093

[30] Oem J.-K. , Lee K.-N. , and Roh I. S. , et al.Identification and Characterization of Rabbit Hemorrhagic Disease Virus Genetic Variants Isolated in Korea, Journal of Veterinary Medical Science. (2009) 71, no. 11, 1519–1523, 10.1292/jvms.001519. 19959905

[31] Fitzner A. , Niedbalski W. , Paprocka G. , and Kęsy A. , Identification of Polish RHDVa Subtype Strains Based on the Analysis of a Highly Variable Part VP60 Gene, Polish Journal of Veterinary Sciences. (2012) 15, no. 1, 10.2478/v10181-011-0109-5. 22708353

[32] Abrantes J. , Lopes A. M. , Dalton K. P. , Parra F. , and Esteves P. J. , Detection of RHDVa on the Iberian Peninsula: Isolation of an RHDVa Strain From a Spanish Rabbitry, Archives of Virology. (2014) 159, no. 2, 321–326, 10.1007/s00705-013-1808-2. 23942953

[33] Mahar J. E. , Read A. J. , and Gu X. , et al.Detection and Circulation of a Novel Rabbit Hemorrhagic Disease Virus in Australia, Emerging Infectious Diseases. (2018) 24, no. 1, 22–31, 10.3201/eid2401.170412. PMC5749467 29260677

[34] Kerr P. J. , Kitchen A. , and Holmes E. C. , Origin and Phylodynamics of Rabbit Hemorrhagic Disease Virus, Journal of Virology. (2009) 83, no. 23, 12129–12138, 10.1128/JVI.01523-09. PMC2786765 19759153

[35] Puggioni G. , Cavadini P. , and Maestrale C. , et al.The New French 2010 Rabbit Hemorrhagic Disease Virus Causes an RHD-Like Disease in the Sardinian Cape Hare (*Lepus capensis* Mediterraneus), Veterinary Research. (2013) 44, no. 1, 10.1186/1297-9716-44-96, 96. PMC3853023 24099575

[36] Wang X. , Xu F. , and Liu J. , et al.Atomic Model of Rabbit Hemorrhagic Disease Virus by Cryo-Electron Microscopy and Crystallography, PLoS Pathogens. (2013) 9, no. 1, 10.1371/journal.ppat.1003132. PMC3547835 23341770

[37] Camarda A. , Pugliese N. , and Cavadini P. , et al.Detection of the New Emerging Rabbit Haemorrhagic Disease Type 2 Virus (RHDV2) in Sicily From Rabbit (*Oryctolagus cuniculus*) and Italian Hare (*Lepus corsicanus*), Research in Veterinary Science. (2014) 97, no. 3, 642–645, 10.1016/j.rvsc.2014.10.008. 25458493

[38] Gall-Reculé G. Le , Lemaitre E. , and Bertagnoli S. , et al.Large-Scale Lagovirus Disease Outbreaks in European Brown Hares (*Lepus europaeus*) in France Caused by RHDV2 Strains Spatially Shared With Rabbits (*Oryctolagus cuniculus*), Veterinary Research. (2017) 48, no. 1, 10.1186/s13567-017-0473-y, 70. PMC5660455 29080562

[39] Velarde R. , Cavadini P. , and Neimanis A. , et al.Spillover Events of Infection of Brown Hares (*Lepus europaeus*) With Rabbit Haemorrhagic Disease Type 2 Virus (RHDV2) Caused Sporadic Cases of an European Brown Hare Syndrome-Like Disease in Italy and Spain, Transboundary and Emerging Diseases. (2017) 64, no. 6, 1750–1761, 10.1111/tbed.12562. PMC5697611 27615998

[40] Velarde R. , Abrantes J. , and Lopes A. M. , et al.Spillover Event of Recombinant *Lagovirus europaeus* /GI.2 Into the Iberian hare (*Lepus granatensis*) in Spain, Transboundary and Emerging Diseases. (2021) 68, no. 6, 3187–3193, 10.1111/tbed.14264. 34324796

[41] Asin J. , Rejmanek D. , and Clifford D. L. , et al.Early Circulation of Rabbit Haemorrhagic Disease Virus Type 2 in Domestic and Wild Lagomorphs in Southern California, USA (2020–2021), Transboundary and Emerging Diseases. (2022) 69, e394–e405, 10.1111/tbed.14315. 34487612

[42] Fitzner A. , Niedbalski W. , and Hukowska-Szematowicz B. , Simultaneous Occurrence of Field Epidemics of Rabbit Hemorrhagic Disease (RHD) in Poland Due to the Co-Presence of Lagovirus Europaeus GI.1 (RHDV)/GI.1a (RHDVa) and GI.2 (RHDV2) Genotypes, Viruses. (2025) 17, no. 10, 10.3390/v17101305, 1305. PMC12568209 41157577

[43] Lopes A. M. , Dalton K. P. , and Magalhães M. J. , et al.Full Genomic Analysis of New Variant Rabbit Hemorrhagic Disease Virus Revealed Multiple Recombination Events, Journal of General Virology. (2015) 96, no. 6, 1309–1319, 10.1099/vir.0.000070. 25626685

[44] Silvério D. , Lopes A. M. , and Melo-Ferreira J. , et al.Insights Into the Evolution of the New Variant Rabbit Haemorrhagic Disease Virus (GI.2) and the Identification of Novel Recombinant Strains, Transboundary and Emerging Diseases. (2018) 65, no. 4, 983–992, 10.1111/tbed.12830. 29430852

[45] Mahar J. E. , Jenckel M. , and Huang N. , et al.Frequent Intergenotypic Recombination Between the Non-Structural and Structural Genes Is a Major Driver of Epidemiological Fitness in Caliciviruses, Virus Evolution. (2021) 7, no. 2, 10.1093/ve/veab080, veab080. PMC8570162 34754513

[46] Bull R. A. , Hansman G. S. , Clancy L. E. , Tanaka M. M. , Rawlinson W. D. , and White P. A. , Norovirus Recombination in ORF1/ORF2 Overlap, Emerging Infectious Diseases. (2005) 11, no. 7, 1079–1085, 10.3201/eid1107.041273. PMC3371806 16022784

[47] Ludwig-Begall L. F. , Mauroy A. , and Thiry E. , Norovirus Recombinants: Recurrent in the Field, Recalcitrant in the Lab—A Scoping Review of Recombination and Recombinant Types of Noroviruses, Journal of General Virology. (2018) 99, no. 8, 970–988, 10.1099/jgv.0.001103. 29906257

[48] Abrantes J. , Droillard C. , and Lopes A. M. , et al.Recombination at the Emergence of the Pathogenic Rabbit Haemorrhagic Disease Virus Lagovirus Europaeus/GI.2, Scientific Reports. (2020) 10, no. 1, 10.1038/s41598-020-71303-4, 14502. PMC7468141 32879332

[49] Cavadini P. , Trogu T. , Velarde R. , Lavazza A. , and Capucci L. , Recombination Between Non-Structural and Structural Genes as a Mechanism of Selection in Lagoviruses: The Evolutionary Dead-End of an RHDV2 Isolated From European Hare, Virus Research. (2024) 339, 10.1016/j.virusres.2023.199257, 199257. PMC10654597 38347757

[50] World Organisation for Animal Health , Rabbit Haemorrhagic Disease (Chapter 3.07.02), The Manual of Diagnostic Tests and Vaccines for Terrestrial Animals, 2021, WOAH.

[51] Frohman M. A. , Dush M. K. , and Martin G. R. , Rapid Production of Full-Length cDNAs From Rare Transcripts: Amplification Using a Single Gene-Specific Oligonucleotide Primer, Proceedings of the National Academy of Sciences. (1988) 85, no. 23, 8998–9002, 10.1073/pnas.85.23.8998. PMC282649 2461560

[52] Cavadini P. , Vismarra A. , and Merzoni F. , et al.Two Decades of Occurrence of Non-Pathogenic Rabbit Lagoviruses in Italy and Their Genomic Characterization, Scientific Reports. (2024) 14, no. 1, 10.1038/s41598-024-79670-y. PMC11589700 39587141

[53] Kumar S. , Stecher G. , Li M. , Knyaz C. , Tamura K. , and Battistuzzi F. U. , MEGA X: Molecular Evolutionary Genetics Analysis Across Computing Platforms, Molecular Biology and Evolution. (2018) 35, no. 6, 1547–1549, 10.1093/molbev/msy096. PMC5967553 29722887

[54] Minh B. Q. , Schmidt H. A. , and Chernomor O. , et al.IQ-TREE 2: New Models and Efficient Methods for Phylogenetic Inference in the Genomic Era, Molecular Biology and Evolution. (2020) 37, no. 5, 1530–1534, 10.1093/molbev/msaa015. PMC7182206 32011700

[55] Kalyaanamoorthy S. , Minh B. Q. , Wong T. K. F. , von Haeseler A. , and Jermiin L. S. , ModelFinder: Fast Model Selection for Accurate Phylogenetic Estimates, Nature Methods. (2017) 14, no. 6, 587–589, 10.1038/nmeth.4285. PMC5453245 28481363

[56] Bouckaert R. , Heled J. , and Kühnert D. , et al.BEAST 2: A Software Platform for Bayesian Evolutionary Analysis, PLoS Computational Biology. (2014) 10, no. 4, 10.1371/journal.pcbi.1003537. PMC3985171 24722319

[57] Martin D. P. , Murrell B. , Golden M. , Khoosal A. , and Muhire B. , RDP4: Detection and Analysis of Recombination Patterns in Virus Genomes, Virus Evolution. (2015) 1, no. 1, 10.1093/ve/vev003, vev003. PMC5014473 27774277

[58] Lole K. S. , Bollinger R. C. , and Paranjape R. S. , et al.Full-Length Human Immunodeficiency Virus Type 1 Genomes From Subtype C-Infected Seroconverters in India, With Evidence of Intersubtype Recombination, Journal of Virology. (1999) 73, no. 1, 152–160, 10.1128/JVI.73.1.152-160.1999. PMC103818 9847317

[59] Kosakovsky Pond S. L. , Posada D. , Gravenor M. B. , Woelk C. H. , and Frost S. D. W. , Automated Phylogenetic Detection of Recombination Using a Genetic Algorithm, Molecular Biology and Evolution. (2006) 23, no. 10, 1891–1901, 10.1093/molbev/msl051. 16818476

[60] Lopes A. M. , Capucci L. , and Gavier-Widén D. , et al.Molecular Evolution and Antigenic Variation of European Brown Hare Syndrome Virus (EBHSV), Virology. (2014) 468–470, 104–112, 10.1016/j.virol.2014.08.002. 25155199

[61] Capucci L. , Frigoli G. , Rønshold L. , Lavazza A. , Brocchi E. , and Rossi C. , Antigenicity of the Rabbit Hemorrhagic Disease Virus Studied by Its Reactivity With Monoclonal Antibodies, Virus Research. (1995) 37, no. 3, 221–238, 10.1016/0168-1702(95)00033-M. 8533459

[62] Abrantes J. , Bertagnoli S. , and Cavadini P. , et al.Comment on Shah et al. Genetic Characteristics and Phylogeographic Dynamics of Lagoviruses, 1988–2021. Viruses 2023, 15, 815, Viruses. (2024) 16, no. 6, 10.3390/v16060927, 927. PMC11209181 38932219

[63] Altschul S. F. , Gish W. , Miller W. , Myers E. W. , and Lipman D. J. , Basic Local Alignment Search Tool, Journal of Molecular Biology. (1990) 215, no. 3, 403–410, 10.1016/S0022-2836(05)80360-2. 2231712

[64] Capucci L. , Scicluna M. T. , and Lavazza A. , Diagnosis of Viral Haemorrhagic Disease of Rabbits and the European Brown Hare Syndrome, Revue Scientifique et Technique de l’OIE. (1991) 10, no. 2, 347–370, 10.20506/rst.10.2.561. 1662098

[65] Podadera A. , Leuthold M. , and Martín-Alonso J. M. , et al.Epitope Mapping of a Neutralizing Antibody Against Rabbit Hemorrhagic Disease Virus GI.2, Veterinary Research. (2025) 56, no. 1, 10.1186/s13567-025-01505-z, 74. PMC11963670 40176146

[66] Lopes A. M. , Almeida T. , and Diz S. , et al.The Potential Role of Scavenging Flies as Mechanical Vectors of Lagovirus Europaeus/GI.2, Virology Journal. (2023) 20, no. 1, 10.1186/s12985-023-02065-4, 103. PMC10224229 37237382

[67] Hrynkiewicz R. , Bębnowska D. , Kauppinen A. , Gadd T. , Piasecki T. , and Niedźwiedzka-Rystwej P. , Occurrence of Lagovirus Europaeus (Rabbit Hemorrhagic Disease Virus) in Domestic Rabbits in Southwestern Poland in 2019: Case Report, Microbiology Spectrum. (2022) 10, no. 6, 10.1128/spectrum.02298-22. PMC9769870 36445093

[68] Merchán T. , Rocha G. , and Alda F. , et al.Detection of Rabbit Haemorrhagic Disease Virus (RHDV) in Nonspecific Vertebrate Hosts Sympatric to the European Wild Rabbit (*Oryctolagus cuniculus*), Infection, Genetics and Evolution. (2011) 11, no. 6, 1469–1474, 10.1016/j.meegid.2011.05.001. 21600308

[69] Calvete C. , Mendoza M. , and Sarto M. P. , et al.Detection of Rabbit Hemorrhagic Disease Virus GI.2/RHDV2/b in the Mediterranean Pine Vole (*Microtus duodecimcostatus*) and White-Toothed Shrew (*Crocidura russula*), Journal of Wildlife Diseases. (2019) 55, no. 2, 467–472, 10.7589/2018-05-124. 30226801

[70] Abade dos Santos F. A. , Pinto A. , and Burgoyne T. , et al.Spillover Events of Rabbit Haemorrhagic Disease Virus 2 (Recombinant GI.4P-GI.2) From Lagomorpha to Eurasian Badger, Transboundary and Emerging Diseases. (2022) 69, no. 3, 1030–1045, 10.1111/tbed.14059. 33683820

[71] Fitzner A. and Niedbalski W. , Phylogenetic Analysis of Rabbit Haemorrhagic Disease Virus (RHDV) Strains Isolated in Poland, Archives of Virology. (2017) 162, no. 10, 3197–3203, 10.1007/s00705-017-3476-0. PMC5602050 28702932

[72] Massardi B. , Genetic and Antigenic Evolution of RNA Viruses: The Case of Rabbit Haemorrhagic Disease Virus, Tesi di Laurea Magistrale, 2010, University of Milan, Faculty of Mathematical, Physical and Natural Sciences.

[73] Leuthold M. M. , Kilic T. , and Devant J. M. , et al.Structural Basis for Rabbit Hemorrhagic Disease Virus Antibody Specificity, Journal of Virology. (2022) 96, no. 22, 10.1128/jvi.01217-22. PMC9682983 36326275

