# Supplementary material for: Detection and Characterisation of The ‘A’ Variant of Rabbit Haemorrhagic Disease (RHDVa, GI.1a) Over a Decade (2013–2023) in Italy
Source: Transbound Emerg Dis. 2026 Sep 30;2026:8762378. doi: 10.1155/tbed/8762378 (PMC13628161; doi:10.1155/tbed/8762378)
Supplement: Supplementary file 1 [file TBED-2026-8762378-s001.zip › S3.docx]

**Figure S3. A)** Maximum likelihood (ML) phylogenetic tree based on the full-length genome alignment (6,851 nt) of RHDV strains. The ML tree was reconstructed using IQ-TREE v2.4.0 under the best-fitting nucleotide substitution model (GTR+F+I+R4), as selected by ModelFinder according to the Akaike Information Criterion (AIC). Branch support was evaluated using 1,000 ultrafast jackknife replicates. The tree was inferred as unrooted and visualised using sequence KC832839_EBHSV as the outgroup. Scale bar represents nucleotide substitutions per site

**Figure S3. B)** Maximum likelihood (ML) phylogenetic tree of the non-structural coding region (5,222 nt) of RHDV strains. Phylogenetic analysis was conducted using IQ-TREE v2.4.0 based on the 5,222-nt non-structural genomic region under the SYM+I+R4 substitution model, identified via ModelFinder (AIC). Branch reliability was assessed using 1,000 ultrafast jackknife replicates. The sequence KC832839_EBHSV serves as the outgroup for visual representation. The scale bar denotes substitutions per site.

**Figure S3. C)** Maximum likelihood (ML) phylogenetic tree of the structural coding region (VP60, 1,629 nt) of RHDV strains. The ML phylogeny was inferred using IQ-TREE v2.4.0 applied to the complete VP60 coding sequence alignment (1,629 nt) under the GTR+F+I+R3 model selected by ModelFinder (AIC). Node support was determined using 1,000 ultrafast jackknife replicates. Sequence KC832839_EBHSV was set as the outgroup for display. The scale bar represents nucleotide substitutions per site.
