# Supplementary material for: Detection and Characterisation of The ‘A’ Variant of Rabbit Haemorrhagic Disease (RHDVa, GI.1a) Over a Decade (2013–2023) in Italy
Source: Transbound Emerg Dis. 2026 Sep 30;2026:8762378. doi: 10.1155/tbed/8762378 (PMC13628161; doi:10.1155/tbed/8762378)
Supplement: Supplementary file 1 [file TBED-2026-8762378-s001.zip › S4.docx]

**Figure S4. A)** Bayesian phylogenetic tree based on the full-length genome alignment (6,851 nt) of RHDV strains. The tree was reconstructed using BEAST v2.7.7 under a strict molecular clock and a Bayesian Skyline coalescent tree prior. Nucleotide substitutions were modelled using the GTR model with gamma-distributed rate heterogeneity among sites (Γ, five categories). Markov chain Monte Carlo (MCMC) chains were run for 10 million generations, sampling every 1,000 generations. Posterior probability values are indicated at the key nodes. The tree is rooted using sequence KC832839_EBHSV as the outgroup for display purposes. Scale bar indicates substitutions per site.

**Figure S4. B)** Bayesian phylogenetic tree of the non-structural coding region (5,222 nt) of RHDV strains. Reconstruction was performed using BEAST v2.7.7 based on the 5,222-nt non-structural gene alignment under a strict molecular clock and a Bayesian Skyline coalescent model. Nucleotide substitution was modelled using the GTR+Γmodel (five rate categories). MCMC chains were run for 10 million generations (sampling frequency: 1/1,000). Bayesian posterior probabilities are shown at major nodes. The scale bar represents nucleotide substitutions per site.

**Figure S4. C)** Bayesian phylogenetic tree of the structural coding region (VP60, 1,629 nt) of RHDV strains. The phylogenetic relationships were inferred using BEAST v2.7.7 based on the 1,629-nt complete VP60 gene alignment under a strict molecular clock and a Bayesian Skyline tree prior with a GTR+Γ substitution model (five rate categories). Node support is given by Bayesian posterior probabilities. The scale bar represents nucleotide substitutions per site.
